# Switch to Aflibercept in the Treatment of Neovascular AMD: Long-Term Results

**DOI:** 10.1155/2017/6835782

**Published:** 2017-10-19

**Authors:** Pedro Neves Cardoso, Ana Fernanda Pinheiro, Jorge Meira, Ana Catarina Pedrosa, Manuel S. Falcão, João Pinheiro-Costa, Fernando Falcão-Reis, Ângela M. Carneiro

**Affiliations:** ^1^Department of Ophthalmology, Centro Hospitalar de São João, University of Porto, Porto, Portugal; ^2^Faculty of Medicine, University of Porto, Porto, Portugal; ^3^Departments of Surgery and Physiology, Faculty of Medicine, University of Porto, Porto, Portugal; ^4^Department of Anatomy, University of Porto, Porto, Portugal

## Abstract

**Purpose:**

To report the long-term clinical outcomes after switching from intravitreal bevacizumab or ranibizumab to aflibercept therapy in eyes with AMD.

**Methods:**

Retrospective analysis of changes in BCVA, SD-OCT image, and frequency of injections after 1, 2, and 3 years of follow-up.

**Results:**

164 eyes were analyzed, 101 eyes switched from bevacizumab (group 1) and 63 from ranibizumab (group 2). One year after the switch, there was an overall nonsignificant mean decrease of 2 ETDRS letters in BCVA. Three years after, there was an overall mean decrease of 7 ETDRS letters, which was statistically significant. A significant improvement in the mean CRT was found at 1, 2, and 3 years. There was a significant decrease in the mean number of injections per year (7.8 to 6.5, *p* < 0.005) between the first and third year.

**Conclusion:**

Aflibercept can be useful in the management of refractory neovascular AMD, with a good morphological response. However, in the long-term, BCVA stabilization was not achieved.

## 1. Introduction

Age-related macular degeneration (AMD) is a chronic degenerative process and is the leading cause of blindness of elderly individuals living in industrialized countries [1, 2]. The neovascular form of AMD is characterized by the presence of choroidal neovascularization (CNV) and its diagnosis is usually based on findings from fundus biomicroscopy, spectral-domain optical coherence tomography (SD-OCT), fluorescein angiography (FA), and indocyanine green angiography (ICGA) [3].

Despite the outstanding advances made by anti-VEGF therapy, persistent fluid or recurrent exudation still occurs [4]. The literature shows that there are two types of patients in which this occurs: nonresponder patients and patients who stop responding during the course of anti-VEGF therapy. In some cases, tachyphylaxis can occur after an initial dose or following a series of small doses [4, 5]. Tachyphylaxis cannot be overcome by increasing the dosage and its mechanism is still not clear. Keane et al. [6] were the first to suggest that possible resistance had appeared after treatment with ranibizumab, whereas other researchers have considered that it may also occur with bevacizumab and as early as after two injections [4, 7, 8]. Gasperini et al. [7] reported that the majority of tachyphylatic patients responded favourably after switching the anti-VEGF.

The advantage of switching between bevacizumab and ranibizumab could be due to differences in molecular size and/or the differing mechanisms of transport through the retina and into the subretinal space. Ranibizumab, a smaller molecule, was found diffusely across the retina after intravitreal injection, while bevacizumab reaches the subretinal space with a different retinal distribution after intravitreal injection [4].

The most recent anti-VEGF agent is aflibercept, a recombinant fusion protein, with (i) a wider spectrum of action, as a result of its higher binding affinity for VEGF-A and VEGF-B and placental growth factors 1 and 2 (PLGF1 and PLGF2) and (ii) a longer half-life in the vitreous (when compared to ranibizumab) [9]. Griffin et al. [10] observed anatomical improvements such as reduction of central retinal thickness and total fluid volume after three aflibercept injections. Kumar et al. [11] found a significant improvement in visual outcomes for treatment-resistant patients who switched to aflibercept. Thus, aflibercept seems to be an effective salvage therapy for neovascular AMD patients who respond poorly to other anti-VEGF drugs [12].

In the past, at the Department of Ophthalmology of Hospital de São João, when the first anti-VEGF drug became available—ranibizumab—it was used to treat the neovascular AMD patients. Later, when bevacizumab was deemed a safe alternative, it became the first-line therapy (it was deemed more cost-effective), and consequently, patients were switched automatically to this drug. Henceforth, ranibizumab became the salvage therapy for patients with refractory or recurrent neovascular AMD, who were under treatment with bevacizumab. However, after May 2013, it was decided that aflibercept would substitute ranibizumab as the salvage molecule in our hospital. Therefore, all patients that were being treated with ranibizumab (because of previous resistance to bevacizumab) were changed to aflibercept.

In a previous paper, we described the short-term results of this therapeutic switch in our center [13].

In the following retrospective analysis, we evaluate the long-term clinical outcome of intravitreal aflibercept therapy in eyes with persistent oedema and recurrent neovascular AMD switched from intravitreal bevacizumab or ranibizumab.

## 2. Patients and Methods

We retrospectively reviewed medical records of all patients with neovascular AMD treated at the Hospital de São João, Porto, Portugal, a tertiary care center, between March 2013 and December 2016.

This interventional and noncomparative study was reviewed and approved by the hospital's Ethics Committee and followed the tenets of the Declaration of Helsinki. Data was collected from patients' charts and analysed from November 2016 to March 2017. All patients under treatment with intravitreal bevacizumab or ranibizumab for neovascular AMD that switched to aflibercept were studied. All patients were followed up continuously at the Hospital de São João and were treated with an as-needed regimen, with monthly assessment, or more recently with a treat and extend regimen (T&E), treatment in accordance with the maximum fluid-free interval.

In our study, the inclusion criteria were (1) the presence of neovascular AMD previously treated with intravitreal bevacizumab or ranibizumab that was switched to intravitreal aflibercept, (2) at least ten ETDRS letters of best-corrected visual acuity (BCVA) before the first intravitreal injection, (3) a minimum of three injections of bevacizumab or ranibizumab before the switch, and (4) at least one year of follow-up after switching the anti-VEGF drug. Exclusion criteria were (1) CNV lesions secondary to causes other than AMD, (2) the presence of −6.00 D or greater myopia, (3) concomitant retinal vascular disorders in the studied eye, cataract surgery, or YAG capsulotomy performed during the follow-up period, and (4) BCVA less than ten ETDRS letters. The fact that these patients had been previously treated with verteporfin or pegaptanib was not an exclusion criterion.

Most patients reviewed had a long history of injections before the switch to aflibercept. Indication for conversion to aflibercept was a failed response to bevacizumab, defined as persistent or recurrent subretinal and/or intraretinal fluid on spectral domain optical coherence tomography (SD-OCT).

One hundred and sixty-four eyes of 134 patients met the inclusion criteria and were analysed. During the follow-up period, BCVA was evaluated using the Early Treatment Diabetic Retinopathy Study (ETDRS) charts. The follow-up involved stereoscopic fundus examination, indocyanine green angiography, fluorescein angiography, and SD-OCT scanning. Only 2 experienced ophthalmologists (Â.M.C. and M.S.F.) performed the first visit's SD-OCT and angiography evaluations.

The CNV lesions were, at that time, classified (by fluorescein angiography) into occult with no classic, predominantly classic, minimally classic, polypoidal choroidal vasculopathy (PCV), or retinal angiomatous proliferation (RAP). The presence of large areas of geographic atrophy, fibrosis, haemorrhage, or retinal pigment epithelium detachments that took on more than half of the lesion's size was not considered a criterion for exclusion from our study.

The patients were followed up with Spectralis HRA-OCT platform (Heidelberg Engineering, Heidelberg, Germany). Foveal thickness measurements were made by the observer on a scan he interpreted as intersecting the fovea and using the caliper tool.

At each follow-up visit, all patients were revaluated by ETDRS chart score, fundoscopic examination, and SD-OCT. Angiographies with fluorescein and indocyanine green were done when there was a significant visual acuity decay and/or in situations of poor response to the current treatment. Retreatment was performed if fluid was present (intraretinal or subretinal) on the SD-OCT scans or if a new macular haemorrhage had developed.

The treatment consisted of an intravitreal injection via pars plana of bevacizumab (1.25 mg), ranibizumab (0.5 mg), or aflibercept (2 mg), in which one is being dependent on the clinical criteria and the time frame in which it occurred. All treatments were performed by an ophthalmologist in the operating room and under full aseptic conditions. It was decided to divide and analyse the patients as two separate groups: patients deemed refractory to bevacizumab (group 1) and patients that were switched from ranibizumab to aflibercept not under clinical criteria but because of an institutional policy board decision (group 2). Patients were considered refractory to bevacizumab when persistent exsudation was present after three or more consecutive monthly bevacizumab injections, regardless of BCVA score. Persistent exudation was considered to be present if there was intraretinal fluid and/or subretinal fluid on SD-OCT images. Patients under therapy with ranibizumab had lesions that were classified as recurrent (exudation suppressed with treatment, but, upon suspension, intraretinal or subretinal fluid recurred).

The main clinical outcomes analysed were variations of BCVA after switching to aflibercept treatment, the retinal morphological response (changes in fluid and foveal thickness on SD-OCT), and the frequency of aflibercept injections after 1, 2, and 3 years of follow-up.

Statistical analysis was performed using SPSS statistical software (version 24.0 for Windows; SPSS Inc., Chicago, IL, USA). Treatment frequency and visual acuity were statistically compared between each treatment regimen using the paired Student *t*-test. Further comparisons between groups were conducted using the two-sample *t*-test. Values in the text will be represented as means ± standard deviation. A *p* value of <0.01 was considered statistically significant.

## 3. Results

### 3.1. Patient and Treatment Characteristics

One hundred and sixty-four eyes of 134 patients met the inclusion criteria and were analysed (Table 1). The mean age of our sample was 75.9 ± 7 years (range: 56–92). Fifty-nine patients (44.0%) were male and 75 (56.0%) were female. The distribution when classified according to angiographic findings was 95 (57.9%) occult, 37 (22.6%) predominantly classic, 11 (6.7%) minimally classic, 14 (8.5%) PCV, and 7 (4.3%) RAP.

One hundred and one eyes (61.6%) were switched to aflibercept because they were refractory to bevacizumab (group 1), and 63 eyes (38.4%) were switched from ranibizumab to aflibercept due to hospital policy change (group 2). Most of the patients analysed had a very long follow-up period and long history of intravitreal treatment. The mean time of treatment before the switch was 28.5 ± 19.1 months overall (18.4 ± 10.8 months for the patients from group 1 and 44.8 ± 18.1 months for the patients from group 2). Most patients of group 2 had a particularly long follow-up before the switch given that they had a previous poor response to bevacizumab which had motivated the first switch to ranibizumab, our salvage molecule in the past.

The mean BCVA in group 1 was 57.4 ± 16.1 letters at baseline and 56.5 ± 16.8 letters at the moment of the therapeutic switch to aflibercept, while in group 2, the mean BVCA was 59.6 ± 15.4 letters at baseline and 56.3 ± 13.9 letters when the switch occurred. The mean central retinal thickness (CRT) at the moment of switch to aflibercept was 406.7 ± 184.4 *μ*m in group 1 and 370.7 ± 163.8 *μ*m in group 2. The mean duration of follow-up after the switch to aflibercept was 29.9 ± 9.0 months overall, 28.0 ± 8.0 months for group 1 and 34.3 ± 8.8 months for group 2. The mean number of aflibercept injections in the overall sample was 15.1 ± 7.3 (range: 3–37), 14.5 ± 7.2 in group 1 and 16.2 ± 7.3 in group 2.

From the 101 eyes in group 1, all 101 had 1 year of follow-up, 66 eyes had 2 years of follow-up, and 22 eyes had 3 years of follow-up.

From the 63 eyes in group 2, all 63 had 1 year of follow-up, 52 eyes had 2 years of follow-up, and 36 eyes had 3 years of follow-up.

### 3.2. Visual Outcomes

The overall mean BCVA before the switch was 56.5 ± 15.7 letters (Table 2). One year after the switch (164 eyes), there was a nonsignificant mean decrease of 2 letters in visual acuity in both groups (group 1: from 56.5 to 54.8 letters, *p* = 0.108; group 2: from 56.3 to 54.3 letters, *p* = 0.127). Two years after the switch (118 eyes), there was a nonsignificant mean decrease of 2 letters in visual acuity in group 1 (from 59.2 to 57.3 letters, *p* = 0.163) and a significant decrease of 5 letters in group 2 (from 56.3 to 51.2 letters, *p* = 0.001). Three years after the switch (58 eyes), there was a nonsignificant mean decrease of 9 letters in visual acuity in group 1 (from 59.9 to 50.8 letters, *p* = 0.016) and a significant decrease of 6 letters in group 2 (from 56.3 to 50.1 letters, *p* = 0.001).

There was a statistically significant decrease in BCVA in the overall sample in both the second and third years (from 57.9 to 54.6 letters, *p* = 0.001 and from 57.6 to 50.3 letters, *p* < 0.001, resp.).

### 3.3. Anatomic Outcomes

CRT was significantly reduced at the end of follow-up in all groups (Table 2). A mean decrease in CRT of 73 *μ*m was noted after one year of aflibercept treatment (from 386.2 to 313.2 *μ*m, *p* < 0.001) with a mean of 7.8 ± 2.6 injections. After the second year and third year, the CRT mean was 280.2 *μ*m and 266.1 *μ*m, respectively.

Qualitatively, 69.5% of the analysed patients in this sample presented a dry SD-OCT image in the last OCT taken (time under treatment with aflibercept ranging from 12 to 44 months).

During the follow-up, no systemic complications or endophthalmitis were registered.

### 3.4. Injection Outcomes

The mean number of aflibercept injections per year was 7.8 during the first year of therapy (Table 2). In the second year, it decreased to 6.8, a statistically significant decrease (*p* < 0.01). In the third year, it further diminished to 6.5 which was also significant (*p* < 0.01).

In the subgroup analysis, the difference was nonsignificant in group 1 (from 8.0 to 7.5 and 7.3, in the second and third years (*p* > 0.01)) and significant in group 2 (from 7.8 to 6.1 and 5.9 in the second and third years (*p* < 0.001)).

## 4. Discussion

Since the introduction of aflibercept, the eyes with neovascular AMD not responding well to injections of ranibizumab or bevacizumab can be switched to treatment with aflibercept.

Our aim was to analyse the clinical outcomes of intravitreal aflibercept therapy in patients with neovascular AMD refractory to bevacizumab and in a group of patients suffering from recurrent disease on treatment with ranibizumab. To do this, we measured (i) anatomical changes like foveal thickness and changes in fluid on SD-OCT, (ii) variations of BVCA after the switch to aflibercept, and (iii) the injection frequency after switching to aflibercept.

In this retrospective study of aflibercept therapy for neovascular AMD that included patients with more than three years of follow-up, foveal anatomic response was the outcome that showed the best response. CRT was significantly reduced in the first year after the switch to aflibercept and that was maintained during the longer follow-up in both groups. And, for the majority of patients (69.5%), the final SD-OCT image had no signs of exsudation.

The overall anatomical success of aflibercept therapy in unresponsive patients may be due to several factors. Firstly, the reported positive effect of aflibercept seems to be related to its pharmacological targets. Aflibercept is a recombinant fusion protein that binds VEGF-A with greater affinity than bevacizumab and ranibizumab [9]. This drug binds other VEGF family members, including VEGF-B, PLGF1, and PLGF2, that are not inhibited by bevacizumab and ranibizumab [14].

Secondly, another plausible explanation relates to tachyphylaxis. As Sarao et al. [14] referenced, a systemic immune response in patients' serum and a local immune response may contribute to the formation of measurable, neutralizing antibodies to bevacizumab or ranibizumab. This may explain the good response observed after switching to another anti-VEGF drug, in this case, aflibercept. After the switch, a lower degree of immunogenicity is expected, leading to sustained benefits in the eyes that had become refractory to bevacizumab and ranibizumab [14]. Another potential contributing factor associated with responsiveness to anti-VEGF therapy is the level of CNV maturation. Mature vessels within CNV have been reported to be covered with pericytes, thereby making the vessels less accessible to therapeutic antibodies [14].

Although there was an improvement in CRT, this study showed that there was not a clear translation of said improvement into the restoration of visual function after a long follow-up period. One year after the switch, there was a nonsignificant mean decrease of 2 letters in visual acuity in both groups, so we can affirm that there was a stabilization of the visual acuity in the first year of follow-up regardless of previous treatment. Nonetheless, there was a statistically significant decrease of 3 letters in the second year and 7 letters in the third year. This could be partly explained by our patients being at a late stage of AMD, with a long history of intravitreal injections with other agents and probably already profound structural changes. The mean time of treatment before the switch to aflibercept was 28.5 ± 19.1 months overall (group 1: 18.4 ± 10.8 months; group 2: 44.8 ± 18.1 months) and after the switch was 29.9 ± 9 months overall (group 1: 28.0 ± 8 months; group 2: 34.3 ± 8.8 months).

Several studies have demonstrated that patients with neovascular AMD improve their baseline visual acuity within the first treatments until they reach a plateau stage in which monthly injections do not result in any further visual improvement [15]. And, it is important to state that, with long follow-ups, a decline in visual acuity is also observed [16–18]. Furthermore, it has been documented as a cause of loss of BVCA, an accumulating effect of long-term neovascularization such as development of retinal gliosis or progression of retinal pigment epithelium atrophy [19].

In the same direction as our results, Spooner et al. [20] evidenced a significant anatomical effect, resulting in CRT thinning, but no associated improvement in visual acuity.

After switching, our patients required fewer aflibercept injections in the third year when compared with the first and second years. The injection intervals increased in all groups throughout the follow-up. These results suggest the possibility of an increase in the treatment intervals and consequently a decrease of injections per year when patients have no fluid in SD-OCT scans at the last visit. Chang et al. [21] showed that aflibercept could maintain visual and anatomical outcomes successfully after spacing treatment to 8 weeks. Naturally, the reduction of the injection frequency lessens the risk of drug- and procedure-related complications and the costs associated with the procedure, periodic by nature.

The limitations of our study are its retrospective design and the noncomparative and consequential nature of the clinical setting. Some biases may arise from the lacking capacity to adjust for patient baseline characteristics and their disease severity.

## 5. Conclusions

Our results show that patients with neovascular AMD, who have had an inadequate response to ranibizumab and/or bevacizumab, benefit from the switch to aflibercept therapy. Despite the patients included in this study being poor responders with limited potential for visual recovery due to the advanced stage of their disease, they still showed an anatomic improvement, with reduction of CRT and retinal exudation and the extension of injection intervals.

Vision did not significantly improve throughout follow-up, but vision stabilization was achieved during the first year which may have an immense impact on the patients' life quality, when compared with the dismal natural history of neovascular AMD.

Switching between different anti-VEGF drugs or combining anti-VEGF therapy with another treatment modality, as PDGF inhibitors, is a therapeutic decision that should be always considered in those with frequent recurrences or refractory patients.

Although the data presented in this study does not unequivocally show it and further studies are necessary, it is probably safe to assume that the switch must occur as soon as possible, to prevent irreversible macular changes that interfere with the potential of future visual acuity recovery or stabilization.

## Figures and Tables

**Table 1:** Patient and treatment characteristics.

Eyes (patients), *n*	164 (134)
Mean age (range), years	75.9 (56–92)
Women, *n* (%)	75 (56.0)
Angiographic classification, *n* (%)	
Occult with no classic	95 (57.9)
Predominantly classic	37 (22.6)
Minimally classic	11 (6.7)
Polypoidal choroidal vasculopathy	14 (8.5)
Retinal angiomatous proliferation	7 (4.3)
Eyes refractory to bevacizumab, *n* (%)	101 (61.6)
Eyes on treatment with ranibizumab, *n* (%)	63 (38.4)
Mean time on therapy ± SD, months	
Prior to switch	28.5 ± 19.1
Aflibercept	29.9 ± 9.0

**Table 2:** Outcomes after switching to aflibercept in patients with neovascular AMD.

	All (*n* = 164, year 1; 118, year 2; 58, year 3)	*p*	Group 1 bevacizumab (*n* = 101, year 1; 66, year 2; 22, year 3)	*p*	Group 2 ranibizumab (*n* = 63, year 1; 52, year 2; 36, year 3)	*p*
BCVA, ETDRS score						
Before switch	56.5 ± 15.7		56.5 ± 16.8		56.3 ± 13.9	
After 1 year	54.6 ± 19.0	0.027	54.8 ± 19.8	0.108	54.3 ± 17.9	0.127
After 2 years	54.6 ± 16.7	0.001	57.3 ± 16.2	0.163	51.2 ± 16.9	0.001
After 3 years	50.3 ± 19.1	<0.001	50.8 ± 22.3	0.016	50.1 ± 17.2	0.001
CRT, *μ*m						
Before switch	386.2 ± 163.7		406.7 ± 184.4		370.7 ± 163.8	
After 1 year	313.2 ± 140.9	<0.001	329.7 ± 152.6	<0.001	287.5 ± 116.8	<0.001
After 2 years	280.2 ± 121.3	<0.001	270.0 ± 107.3	<0.001	293.1 ± 137.1	<0.001
After 3 years	266.1 ± 123.5	<0.001	259.4 ± 85.7	<0.001	269.7 ± 140.9	<0.001
Injections per year, *n*						
In the first year	7.8 ± 2.6		8.0 ± 2.6		7.8 ± 2.1	
In the second year	6.8 ± 3.5	0.007	7.5 ± 3.5	0.219	6.1 ± 3.2	<0.001
In the third year	6.5 ± 3.1	0.002	7.3 ± 2.8	1	5.9 ± 3.1	<0.001

Outcomes are expressed as mean values ± SD.
